# A Peptide Meets a Radionuclide to Combat a Rare Tumor

**DOI:** 10.31486/toj.20.0098

**Published:** 2021

**Authors:** Katharine E. Thomas, J. Philip Boudreaux, Ramcharan Thiagarajan, Andrew Marsala, Brianne A. Voros, Robert A. Ramirez

**Affiliations:** ^1^ Neuroendocrine Tumor Clinic, Ochsner Clinic Foundation, Kenner, LA; ^2^ Department of Hematology/Oncology, Louisiana State University Health Sciences Center, New Orleans, LA; ^3^ Department of Surgery, Louisiana State University Health Sciences Center, New Orleans, LA; ^4^ Department of Radiology, Division of Interventional Radiology, Ochsner Clinic Foundation, New Orleans, LA; ^5^ Department of Internal Medicine, Division of Hematology/Oncology, Ochsner Clinic Foundation, New Orleans, LA

**Keywords:** *Carcinoma–neuroendocrine*, *gallium Ga 68 dotatate*, *lutetium-177*, *receptors–somatostatin*

## Abstract

**Background:** Neuroendocrine carcinomas (NECs) are rare malignancies with limited treatment options beyond surgery. Peptide receptor radionuclide therapy (PRRT) is a process by which a somatostatin analog (octreotate) is combined with a chelator (DOTA) and a radionuclide (lutetium-177 [^177^Lu-dotatate]). This therapy targets receptors on neuroendocrine cells, causing internalization of the radionuclide by the tumor cell, which results in cellular damage and apoptosis.

**Case Report:** We describe the clinical and therapeutic course of a 69-year-old male with a metastatic rectal NEC in whom progressive disease was noted after multiple therapies were attempted. After PRRT with ^177^Lu-dotatate, the patient was asymptomatic and demonstrated a near-complete radiologic response.

**Conclusion:** This case illustrates that treatment with PRRT may improve the outcome of patients with metastatic rectal NEC. Our case highlights the importance of further research into the use of PRRT in patients with a Ki-67 <55% and uptake on somatostatin receptor imaging.

## INTRODUCTION

Neuroendocrine tumors (NETs) are rare malignancies that increased 6.4-fold between 1973 (1.09 per 100,000) and 2012 (6.98 per 100,000).^1^ These malignancies occur most commonly in the gastrointestinal tract. Beyond surgery, treatment options are limited to chemotherapy. In patients with high-grade, poorly differentiated (PD) tumors, termed neuroendocrine carcinomas (NECs), therapeutic options are even more limited.

Peptide receptor radionuclide therapy (PRRT) provides another therapeutic option for patients with advanced, progressive, somatostatin receptor (SSTR)–positive NETs of the midgut.^2^ By combining a somatostatin analog (SSA) (octreotate) with a chelator (DOTA) and a radionuclide (lutetium-177 [^177^Lu-dotatate]), PRRT takes advantage of the SSTRs that are expressed on the surface of most NETs.^3^ This therapy targets the SSTRs on NET cells, allowing internalization of the radionuclide by the tumor cell. Once inside the tumor cell, beta emission from the radionuclide results in cellular damage.^3^

We describe a patient with a metastatic rectal NEC who had a near-complete response with ^177^Lu-dotatate treatment.

## CASE REPORT

**Figure 1.**
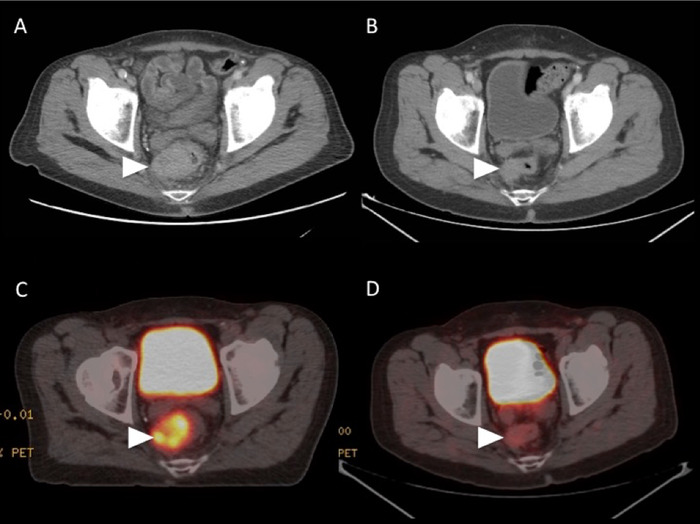
(A) Axial pretreatment contrast-enhanced computed tomography (CT) at the level of the rectum demonstrates the primary rectal tumor (arrowhead). (B) Axial contrast-enhanced CT obtained 8 weeks after completion of peptide receptor radionuclide therapy (PRRT) demonstrates a significant decrease in the size of the primary rectal tumor (arrowhead). (C) Axial fused pretreatment gallium-68 dotatate positron emission tomography (PET)/CT scan demonstrates uptake within the somatostatin receptor-rich primary rectal tumor (arrowhead). (D) Axial fused gallium-68 dotatate PET/CT scan obtained 8 weeks after completion of PRRT demonstrates near-resolution of tracer uptake within the primary rectal tumor (arrowhead).

**Figure 2.**
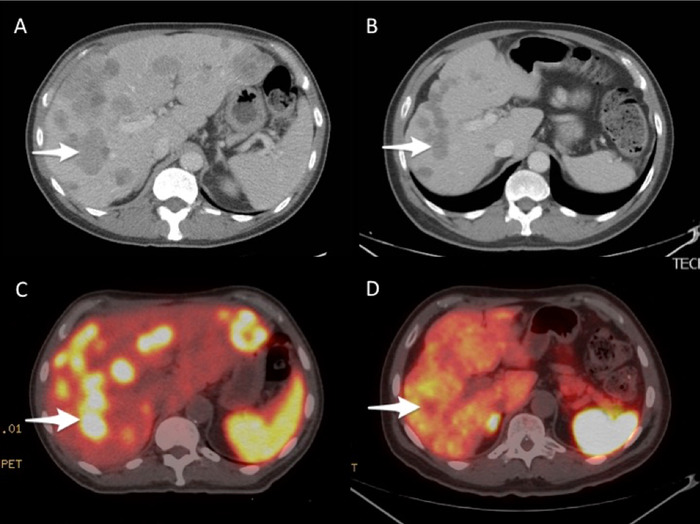
(A) Axial pretreatment contrast-enhanced computed tomography (CT) at the level of the right portal vein demonstrates innumerable hepatic metastatic lesions with index lesion (arrow). (B) Axial contrast-enhanced CT obtained 8 weeks after completion of peptide receptor radionuclide therapy (PRRT) demonstrates a decrease in the size of all lesions, including the index lesion (arrow). (C) Axial fused pretreatment gallium-68 dotatate positron emission tomography (PET)/CT scan demonstrates somatostatin receptor-rich hepatic metastatic lesions with index lesion (arrow). (D) Axial fused gallium-68 dotatate PET/CT scan obtained 8 weeks after completion of PRRT demonstrates resolution of tracer uptake within the metastatic lesions, including the index lesion (arrow).

A 69-year-old male with a history of a metastatic rectal NEC was referred to our center for evaluation. The patient had been diagnosed in October 2014 after developing rectal bleeding. Colonoscopy had shown a fungating, infiltrative, ulcerating, nonobstructive mass measuring 3 cm in length within the rectum. Pathology identified a PD NEC. The tumor was present within the lamina propria and muscularis mucosa, no small-cell features were seen, and the Ki-67 proliferative index was 40%. Computed tomography (CT) showed asymmetric wall thickening involving approximately 50% of the circumference of the rectum and enlarged adjacent perirectal lymph nodes abutting the mesorectal fascia (Figure 1A). CT also revealed innumerable hepatic masses, bilateral pulmonary nodules, and a sclerotic lesion in the right ilium (Figure 2A). Fluorodeoxyglucose positron emission tomography (PET) CT confirmed uptake in multiple liver metastases, which were too numerous to count, as well as uptake in the rectal neoplasm and perirectal lymph node. In January 2015, the patient began treatment with carboplatin area under the curve 5 and etoposide 100 mg/m^2^ every 3 weeks and completed 6 cycles of therapy. Treatment was interrupted because surgery was needed for an expanding meningioma. Scans revealed stable disease, and the patient was judged to be a candidate for prophylactic cranial irradiation. The patient underwent 25 Gy in 10 fractions in August 2015.

Because of progressive disease in the liver, the patient was started on topotecan 1.5 mg/m^2^ on days 1 through 5 every 3 weeks in October 2015. Following the first cycle of treatment, he developed symptomatic hypoglycemia, and insulin was elevated at 67.7 μU/mL (reference range, 0-30 μU/mL). Hypoglycemia was hypothesized to be related to his metastatic liver disease, and the patient underwent transarterial chemoembolization (TACE). Symptoms improved, and he started a second cycle of topotecan in December 2015. In January 2016, the patient started combination capecitabine 750 mg/m^2^ twice daily on days 1 through 14 of a 28-day cycle and temozolomide 200 mg/m^2^ on days 10 through 14 of a 28-day cycle (CAPTEM), along with lanreotide, and was noted to have uptake on somatostatin scintigraphy.

The patient underwent repeat TACE in July 2016 and continued CAPTEM until November 2016 when he was noted to have progression in the liver. In December 2016, the patient was started on treatment with fluorouracil (5-FU) 400 mg/m^2^ followed by a 2,400 mg intravenous infusion over 2 days, leucovorin 400 mg/m^2^, and oxaliplatin 85 mg/m^2^ every 2 weeks (FOLFOX). The patient's disease remained stable until November 2017, when he again had progression in the liver. In December 2017, the patient was started on ipilimumab 1 mg/kg for 30 minutes daily on day 1 and nivolumab 3 mg/kg for 30 minutes daily on days 1, 15, and 29 through a clinical trial. Following 4 cycles of treatment, the patient was noted to have progression of disease and declining performance status. The last treatment was in May 2018.

**Figure 3.**
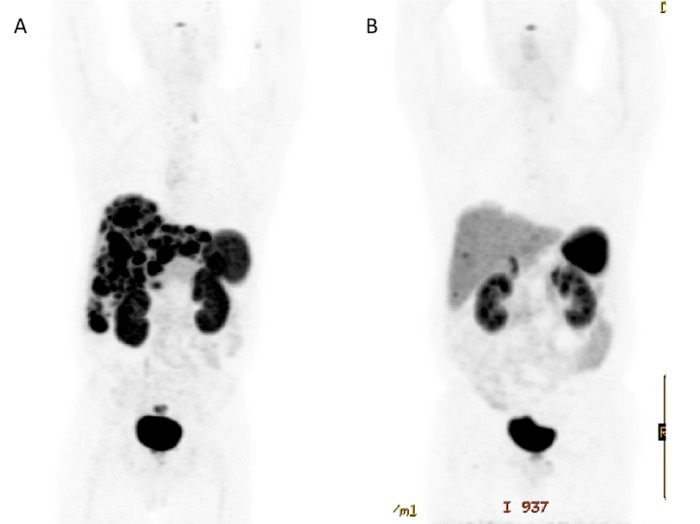
(A) Pretreatment gallium-68 dotatate positron emission tomography (PET) scan demonstrates burden of hepatic metastatic disease, with primary rectal tumor obscured by tracer in the urinary bladder. (B) Gallium-68 dotatate PET scan obtained 8 weeks after completion of PRRT demonstrates near-resolution of abnormal tracer uptake.

The patient sought follow-up in our clinic in May 2018. At that time, he was extremely symptomatic with fatigue, weight loss, and abdominal and rectal pain, and he required assistance to ambulate. After nutritional support and pain control, he was judged to be a candidate for further treatment. Gallium-68 dotatate PET/CT scan in June 2018 showed uptake in diffuse metastatic disease throughout the liver causing hepatomegaly (Figure 3A). Uptake in metastatic lymph nodes in the left lower neck, mediastinum, and rectum was also noted. Axial fused pretreatment gallium-68 dotatate PET/CT scan demonstrated SSTR-rich primary rectal tumor and hepatic metastatic lesions with index lesion (Figures 1C and 2C).

In August 2018, the patient was initiated on ^177^Lu-dotatate and received 200 mCi. He repeated this treatment every 8 weeks for 4 cycles. During this time, his symptoms improved, and he gained weight. By February 2019, when he received his final cycle, the patient was completely asymptomatic. Repeat imaging that included a gallium-68 dotatate PET/CT scan in April 2019 revealed a near-complete response to therapy (Figures 1D, 2D, and 3B). CT of the abdomen and pelvis also revealed partial response (Figures 1B and 2B). A subsequent scan showed responsive disease.

## DISCUSSION

The United States Food and Drug Administration approved ^177^Lu-dotatate in 2018 based on data from the NETTER (Neuroendocrine Tumors Therapy)-1 trial.^2^ The NETTER-1 trial randomized 229 patients with low- and intermediate-grade midgut NETs who had progressed on an SSA to either high-dose octreotide 60 mg every 4 weeks or ^177^Lu-dotatate 200 mCi every 8 weeks for a total of 4 doses with a primary endpoint of progression-free survival (PFS). The results showed that ^177^Lu-dotatate was superior with a median PFS not yet reached in the ^177^Lu-dotatate group and 8.4 months in the high-dose octreotide group (hazard ratio 0.21, 95% CI 0.13-0.33; *P*<0.0001). Responses occurred in 18% of patients, with the majority of patients having stable disease.

Our patient differed from patients in the NETTER-1 study, as he was classified as having an NEC (Ki-67 40%) with PD morphology, and he had a hindgut tumor. The data on high-grade tumors are scarce, and thus, PRRT is not currently a recommended treatment for NEC.^4^ The majority of research on the effectiveness of PRRT has been in low- or intermediate-grade NETs because of the demonstrated inverse relationship between grade and SSTR expression.^5-7^ As previously described, PRRT uses the SSTRs to gain internal access to the cell; therefore, the more SSTRs that a tumor cell expresses, the more successful PRRT is. The current (2019) National Comprehensive Cancer Network treatment guidelines for PD NECs state that somatostatin scintigraphy is not recommended in the evaluation of NECs unless evidence suggests well-differentiated morphology.^4^ Contrary to the current guidelines, however, studies have demonstrated high SSTR expression in certain patients with NEC, despite concomitant PD morphology.^8,9^ For example, Welin et al reported that 62% of patients with NECs had a positive SSTR expression, and Binderup et al described 69% of patients with PD NECs as SSTR positive during their initial evaluation.^8,9^ Congruently, our patient demonstrated SSTR expression and an excellent response to PRRT, despite both a high proliferation rate and PD morphology.

Our patient's response was in line with a patient described by Garske and collegues.^10^ The patient with an NEC of unknown primary with liver metastasis and a Ki-67 10% to 50% had a successful response to PRRT after progression of disease on 2 chemotherapies.^10^ Similarly, our patient was pretreated with multiple lines of chemotherapy, with the addition of liver-directed therapy and immunotherapy.

The current first-line treatment for unresectable NEC is chemotherapy using a platinum-based treatment in combination with etoposide.^4^ The NORDIC (Neuro-Ophthalmology Research Disease Investigator Consortium) NEC trial demonstrated a median overall survival (OS) of 11 months and a median PFS of 4 months in patients when first-line chemotherapy was used in NECs.^11^ Thang et al conducted a recent (2018) retrospective study examining PRRT in NEC and reported more favorable results than the NORDIC NEC trial, with a median OS of 19 months and a median PFS of 9 months in patients who underwent PRRT.^12^ Patients with a Ki-67 <55% did remarkably better with PRRT (46 months vs 14 months) compared to chemotherapy treatment; however, those with a Ki-67 ≥55% had a shorter OS with PRRT compared to treatment with chemotherapy (7 months vs 10 months, respectively).^11,12^ In line with the results reported by Thang and colleagues, Carlsen et al conducted a retrospective cohort study of 149 patients with NEC treated with PRRT and found that those with a Ki-67 21% to 54% experienced longer PFS and OS compared to patients with a Ki-67 ≥55% (16 vs 6 months [*P*<0.001] and 31 vs 9 months [*P*<0.001], respectively).^13^ Parallel to our patient, the majority of subjects examined by Thang et al and Carlsen et al failed prior treatment with chemotherapy (79% and 69.8%, respectively) but responded favorably to PRRT.^12,13^

^177^Lu-dotatate is indicated for SSTR-positive gastroenteropancreatic NETs, including foregut, midgut, and hindgut in adults.^4^ As of October 2020, no grade is specified as a cutoff. Although the NETTER-1 study did not include these patients, considering this treatment for patients with widespread SSTR-positive disease is reasonable.

Our patient demonstrated an excellent response to PRRT, perhaps because of his lower proliferation rate, in spite of having PD morphology. These results demonstrate that, conceivably, PRRT is a more advantageous therapy than chemotherapy for patients with NEC who have a proliferation rate <55%.

Not only did our patient have a dramatic radiologic improvement, but he also improved clinically and became symptom-free. When compared to chemotherapy, PRRT appears to be tolerated well with less adverse effects. The safety of ^177^Lu-dotatate was studied in 504 patients with NETs and was found to produce digestive (such as diarrhea, vomiting, and abdominal pain) and hematologic (neutropenia, anemia, thrombocytopenia [World Health Organization grades 3 or 4]) side effects in 25% and 3.6% of administrations, respectively.^14^ Serious adverse effects, including myelodysplastic syndrome and nonfatal liver toxicity, were reported in <1% of patients.^14^ The side effect profile of PRRT illustrates that ^177^Lu-dotatate may serve as a well-tolerated therapy for patients with NEC.

## CONCLUSION

Our case accentuates the importance of further investigation of PRRT in NEC, specifically in patients with a Ki-67 <55% and uptake on SSTR imaging.

## Notes

*This article meets the Accreditation Council for Graduate Medical Education and the American Board of Medical Specialties Maintenance of Certification competencies for Patient Care and Medical Knowledge.*

## References

[1] Dasari A, Shen C, Halperin D, Trends in the incidence, prevalence, and survival outcomes in patients with neuroendocrine tumors in the United States. JAMA Oncol. 2017;3(10):1335-1342. doi: 10.1001/jamaoncol.2017.0589 28448665 PMC5824320

[2] Strosberg J, El-Haddad G, Wolin E, et al; NETTER-1 Trial Investigators. Phase 3 trial of ^177^Lu-Dotatate for midgut neuroendocrine tumors. N Engl J Med. 2017;376(2):125-135. doi: 10.1056/NEJMoa1607427 28076709 PMC5895095

[3] Hirmas N, Jadaan R, Al-Ibraheem A. Peptide receptor radionuclide therapy and the treatment of gastroentero-pancreatic neuroendocrine tumors: current findings and future perspectives. Nucl Med Mol Imaging. 2018;52(3):190-199. doi: 10.1007/s13139-018-0517-x 29942397 PMC5995780

[4] Neuroendocrine and adrenal tumors version 1.2019. National Comprehensive Cancer Network. 2019. Updated June 2019. Accessed August 28, 2019. http://www.arupconsult.com/reference/nccn-neuroendocrine-and-adrenal-tumors-version-12019"> 10.6004/jnccn.2018.0056 29891520

[5] Ezziddin S, Logvinski T, Yong-Hing C, Factors predicting tracer uptake in somatostatin receptor and MIBG scintigraphy of metastatic gastroenteropancreatic neuroendocrine tumors. J Nucl Med. 2006;47(2):223-233. 16455627

[6] Ezziddin S, Khalaf F, Vanezi M, Outcome of peptide receptor radionuclide therapy with ^177^Lu-octreotate in advanced grade 1/2 pancreatic neuroendocrine tumours. Eur J Nucl Med Mol Imaging. 2014;41(5):925-933. doi: 10.1007/s00259-013-2677-3 24504504

[7] Kayani I, Bomanji JB, Groves A, Functional imaging of neuroendocrine tumors with combined PET/CT using 68Ga-DOTATATE (DOTA-DPhe1,Tyr3-octreotate) and 18F-FDG. Cancer. 2008;112(11):2447-2455. doi: 10.1002/cncr.23469 18383518

[8] Welin S, Sorbye H, Sebjornsen S, Knappskog S, Busch C, Öberg K. Clinical effect of temozolomide-based chemotherapy in poorly differentiated endocrine carcinoma after progression on first-line chemotherapy. Cancer. 2011;117(20):4617-4622. doi: 10.1002/cncr.26124 21456005

[9] Binderup T, Knigge U, Loft A, Functional imaging of neuroendocrine tumors: a head-to-head comparison of somatostatin receptor scintigraphy, 123I-MIBG scintigraphy, and 18F-FDG PET. J Nucl Med. 2010;51(5):704-712. doi: 10.2967/jnumed.109.069765 20395333

[10] Garske U, Sandström M, Johansson S, Lessons on tumour response: imaging during therapy with (177)Lu-DOTA-octreotate. A case report on a patient with a large volume of poorly differentiated neuroendocrine carcinoma. Theranostics. 2012;2(5):459-471. doi: 10.7150/thno.3594 22768026 PMC3360199

[11] Sorbye H, Welin S, Langer SW, Predictive and prognostic factors for treatment and survival in 305 patients with advanced gastrointestinal neuroendocrine carcinoma (WHO G3): the NORDIC NEC study. Ann Oncol. 2012;24(1):152-160. doi: 10.1093/annonc/mds276 22967994

[12] Thang SP, Lung MS, Kong G, Peptide receptor radionuclide therapy (PRRT) in European Neuroendocrine Tumour Society (ENETS) grade 3 (G3) neuroendocrine neoplasia (NEN) - a single-institution retrospective analysis. Eur J Nucl Med Mol Imaging. 2018;45(2):262-277. doi: 10.1007/s00259-017-3821-2 28894897

[13] Carlsen EA, Fazio N, Granberg D, Peptide receptor radionuclide therapy in gastroenteropancreatic NEN G3: a multicenter cohort study. Endocr Relat Cancer. 2019;26(2):227-239. doi: 10.1530/ERC-18-0424 30540557

[14] Kwekkeboom DJ, de Herder WW, Kam BL, Treatment with the radiolabeled somatostatin analog [177 Lu-DOTA 0,Tyr3]octreotate: toxicity, efficacy, and survival. J Clin Oncol. 2008;26(13):2124-2130. doi: 10.1200/JCO.2007.15.2553 18445841

